# Mannose-binding lectin concentrations in people living with HIV/AIDS infected by HHV-8

**DOI:** 10.1186/s12865-018-0284-6

**Published:** 2019-01-03

**Authors:** Viviane Martha Santos de Morais, Juliana Prado Gonçales, Georgea Gertrudes de Oliveira Mendes Cahú, Tania Regina Tozetto-Mendoza, Maria Rosângela Cunha Duarte Coêlho

**Affiliations:** 1 Virology Division, Laboratory of Immunopathology Keizo Asami (LIKA), Federal University of Pernambuco, Recife, Pernambuco Brazil; 2 Laboratory of Virology (LIM52), Institute of Tropical Medicine of São Paulo, University of São Paulo, São Paulo, Brazil; 3 Departament of Physiology and Pharmacology, Center of Biological Sciences, Federal University of Pernambuco, Recife, Pernambuco Brazil; 4 Setor de Virologia do Laboratório de Imunopatologia Keizo Asami, Universidade Federal de Pernambuco, Cidade Universitária, P.O. Box: Av. Prof. Moraes Rego, 1235, Recife, PE 50670-901 Brazil

**Keywords:** HIV/HHV8 coinfection, MBL, Deficient concentrations, Human herpesvirus 8

## Abstract

**Background:**

Mannose-binding lectin (MBL) plays an important role in the innate immune response by activating the complement system via the lectin pathway, and it has been studied in several viral infections; however, the influence of MBL in PLWHA infected with HHV-8 is unknown. The objective of this study was to verify the association of MBL deficient plasma concentrations in HIV/HHV-8 coinfected and HIV monoinfected patients and to correlate these concentrations with HIV viral load and CD4 counts in both groups.

**Results:**

This was an analytical study of case-controls consisting of PLWHA monitored at the medical outpatient of Infectious and Parasitic Diseases of the clinical hospital in the Federal University of Pernambuco. Plasma concentrations of MBL were obtained by an enzyme-linked immunosorbent assay (ELISA) using a commercial Human Mannose Binding Lectin kit (MyBioSource, Inc.) that was performed according to the manufacturer’s guidelines, with values < 100 ng/ml considered deficient. A total of 245 PLWHA samples were analysed; 118 were HIV/HHV-8 coinfected and 127 were HIV monoinfected; 5.1% (6/118) of the coinfected patients and 3.2% (4/127) of the monoinfected patients (*p* = 0.445) were considered plasma concentration deficient. The median of the plasma concentrations of MBL in the coinfected patients was 2803 log_10_ ng/ml and was 2.959 log_10_ ng/ml in the monoinfected patients (*p* = 0.001). There was an inverse correlation between the plasma concentrations of MBL and the HIV viral load in both groups, but no correlation with the CD4 count.

**Conclusions:**

Although the plasma concentrations considered deficient in MBL were not associated with HHV-8 infection in PLWHA, the coinfected patients showed lower MBL concentrations and an inverse correlation with HIV viral load, suggesting that there may be consumption and reduction of MBL due to opsonization of HIV and HHV-8, leading to the reduction of plasma MBL and non-accumulation in the circulation.

## Background

The incidence of Kaposi’s sarcoma (KS) associated with human herpesvirus 8 (HHV-8) has increased in people living with HIV/AIDS (PLWHA), with a more aggressive clinical course and progression to death [1–6]. KS is one of the most common cancers in PLWHA, even when the individuals are treated with antiretroviral therapy (ART) and have an undetectable HIV viral load and CD4 + T cell counts that are above 350 cells/mm^3^ [7–9].

The control of HHV-8, just as in the early stages of the development of KS, is mediated by the innate and adaptive immunity [10–12]. In this context, mannose-binding lectin (MBL) plays a key role in innate immunity as a standard recognition receptor, binding with a high affinity to the residue patterns of carbohydrates present on the surface of viruses or virus-infected cells, especially when the humoral immunity is not fully functional, such as in childhood or in immunosuppressed or immunocompromised populations [13–15]. Thus, MBL contributes to the defence of the innate immune system by initiating the activation of the lectin-complement pathway, which promotes opsonophagocytosis, modulates inflammation and induces cellular lysis [16–18].

Regarding PLWHA, some studies have shown an association between MBL plasma concentration deficiency and HIV infection or a more rapid disease progression [19–23], and others found no association with this infection, the HIV viral load or the CD4 count [18, 24]. However, the influence of MBL on HHV-8 infection is not known, but for other herpes viruses, studies suggest that MBL deficiency may be a risk factor for the symptomatic development of human herpesvirus 2 (HHV-2) [25] and for cytomegalovirus reactivation (CMV) [14, 26]. Although MBL plays an important role in the innate immune response, it is still unknown whether a deficiency in the MBL plasma concentration is associated with HHV-8 infection in PLWHA. In view of the above, the objective of this study was to verify the association of MBL deficient plasma concentrations in HIV/HHV-8 coinfected and HIV monoinfected patients and correlate the concentration with the HIV viral load and CD4 counts in both groups.

## Results

We analysed 245 samples from PLWHA, including 118 HIV/HV-8 coinfected patients, with a mean age of 42.5 (± 11.8), and 127 patients monoinfected with HIV, with mean age of 42.8 (± 10.6), and the majority of these patients were males 71% (84/118) and 59% (75/127), respectively. The median TCD4 count was 2.713 log_10_ cells/mm^3^ (1.301–3.224) in the coinfected patients and 2.736 log_10_ cells /mm^3^ (1.176–3.269) in the monoinfected patients (*p* = 0.872). The HIV viral load was detectable in 42% (50/118) of the coinfected patients and in 44% (56/127) in the monoinfected patients, with a median of 2.057 log_10_ copies/ml (1.672–5.539) and 2.363 log_10_ copies/ml (1.602–6.273), respectively, with *p* = 0.595.

**Fig. 1:**
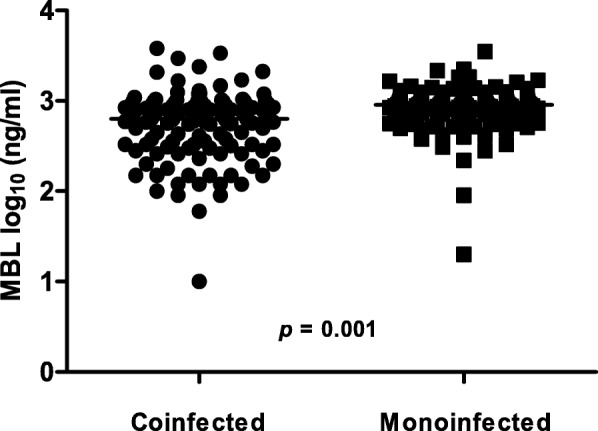
Distribution and median of MBL plasma concentrations in HIV/HHV8 coinfected and HIV monoinfected patients. The median of the coinfected patients was 2.803 log_10_ ng/ml (1.0–3.581; CI: 2.650–2.792), and in the monoinfected, it was 2.959 log_10_ ng/ml (1.301–3.545; CI: 2.877–2.964), which was statistically significant (*p* = 0.001) using the Mann-Whitney test

The plasma concentrations were considered deficient in 5.1% (6/118) of the coinfected patients and in 3.2% (4/127) of the monoinfected patients, with a *p* value of 0.445 (OR = 1.647; IC = 0.453–5.989). However, the MBL plasma concentration median were significantly lower in the coinfected patients, as shown in Fig. 1.

Table 1 shows the median MBL plasma concentrations according to the clinical variables in the coinfected and monoinfected patients.

**Table 1:** Median plasma concentrations of MBL according to sex, age, HIV viral load and CD4 count in HIV/HHV-8 coinfected and HIV monoinfected patients

	Median of MBL (log_10_ ng/ml) (range)^1^				*p-*value^2^
Variables	Coinfected	N^3^	Monoinfected	N^3^	
Sex					
Male	2.806 (1.778–3.581)	84	2.968 (2.342–3.348)	75	0.000
Female	2.778 (1.000–3.378)	34	2.947 (1.301–3.545)	52	0.007
Age					
≥ 40	2.806 (1.000–3.581)	68	2.949 (1.301–3.545)	78	0.005
< 40	2.796 (1.954–3.471)	50	2.987 (1.954–3.348)	49	0.000
HIV viral load					
Detectable	2.752 (1.000–3.318)	50	2.949 (1.301–3.545)	56	0.001
Undetectable	2.874 (1.778–3.581)	68	2.961 (1.954–3.348)	71	0.377
CD4 counts					
≥ 200	2.823 (1.778–3.581)	98	2.964 (1.954–3.545)	115	0.001
< 200	2.586 (1.000–3.167)	20	2.926 (1.301–3.093)	12	0.034

^1^Minimum and maximum values are referenced in bracket; ^2^ *p*-value for the Mann-Whitney test; ^3^ N = sample size

**Fig. 2:**
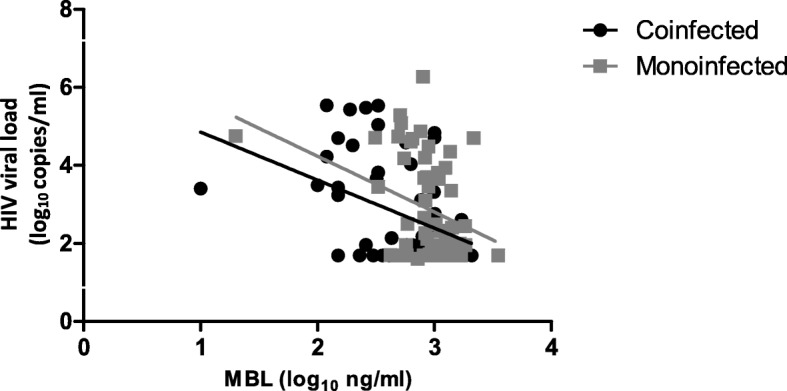
Spearman correlation between HIV viral load and MBL plasma concentration in HIV/HHV-8 coinfected (*n* = 50; *p* = 0.018; *r* = − 0.333) and HIV monoinfected patients (*n* = 56; *p* = 0.031; *r* = − 0.289)

Among the coinfected and monoinfected patients, there was a negative correlation between the HIV viral load and the MBL plasma concentration, as shown in Fig. 2.

**Fig. 3:**
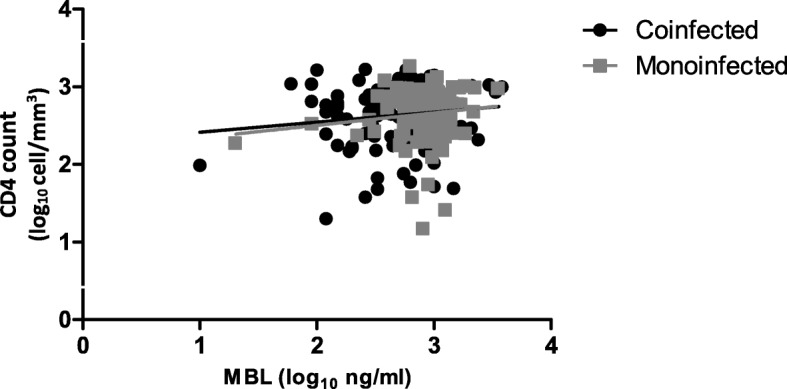
Spearman correlation between the CD4 counts and MBL plasma concentration in the HIV/HHV-8 coinfected (*n* = 118; *p* = 0.346; *r* = 0.087) and HIV monoinfected patients (*n* = 127; *p* = 0.132; *r* = − 0.134)

Figure 3 shows the Spearman correlation between the CD4 count and the MBL plasma concentration in the HIV/HHV-8 coinfected and HIV monoinfected patients.

## Discussion

MBL plays an important role in the innate immune response by activating the complement system via the lectin pathway in an antibody-independent mechanism [15, 17, 23]. This protein has been studied in several infections [26–30]; however, this is the first study to evaluate the plasma concentrations of functional MBL in PLWHA coinfected with HHV-8 and correlate the concentrations with HIV viral load and CD4 counts.

The study population did not differ in age and sex when compared to other studies evaluating PLWHA infected with HHV-8 [4, 8, 31–33]. Similarly, the median HIV viral load and TCD4 count did not show a statistically significant difference between the HIV/HHV-8 coinfected and the HIV monoinfected patients, corroborating works that also evaluated these clinical characteristics [31, 32, 34].

It is still unknown how MBL aids in the control or elimination of HHV-8 infection; however, in relation to HIV infection, studies show that MBL binds to HIV gp120 glycoprotein, helping to clear this virus through the activation of the complement system [22, 35–38]. In addition, when MBL is bound to HIV it can also be eliminated from the circulation by the C1q receptor, which has a structural and functional affinity for MBL [30, 39, 40], suggesting the consumption and reduction of MBL during HIV infection [26, 30].

In relation to the *Herpesviridae* family there are few studies performed. The data of a research show that MBL modulates the response to HSV-2 in mice by affecting neutralization of the virus. Furthermore, reported that the frequency of the MBL deficient (< 100 ng/ml) was higher in the symptomatic group (people with recurrent HSV-2 infections) [25]. In other research the MBL deficient levels were linked to CMV reactivation after lung transplantation [26]. These authors suggest that lack of MBL mediated complement activation increases susceptibility to infections by these viruses.

Studies use different cut-off points, including < 100, < 300 and < 500 ng/ml, to define MBL deficient plasma concentrations [14, 18, 25, 26, 30]. In our study, we defined deficient concentrations as < 100 ng/ml and found no association with HHV-8 infection in PLWHA, the HIV viral load or the CD4 count; however, the median of these concentrations was significantly lower in the HIV/HHV-8 coinfected patients compared to the HIV monoinfected patients. However, the median concentrations found in both groups were higher than the values considered deficient by these authors [14, 18, 19, 25, 26, 30]. A possible explanation for the lower concentrations of MBL in coinfected patients would be the consumption and reduction of this protein, involving the opsonization of HIV and HHV-8, leading to the reduction of plasma MBL and its non-accumulation in the circulation [26].

On the other hand, considering the cut-off point established by Manuel et al. (2007) to characterize the deficient plasma concentration of MBL (< 500 ng/ml), in our study, four coinfected patients who developed KS showed a deficient MBL concentration 2.519 log_10_ ng/ml (data not shown). These same four patients were genetically characterized for the polymorphisms − 550, − 221 and exon 1 of the MBL2 gene in the research conducted by Morais et al. (2018), the authors reported that these coinfected were characterized as intermediate expression haplotypes for the production of the MBL protein, being three HYA/LXA and one LYA/LYO [41]. Because of these results, studies related to MBL plasma concentrations and MBL2 gene polymorphisms become essential and may help to understand the role of MBL in the development of KS in HIV/HHV-8 coinfected patients.

In relation to CD4, the counts above or below 200 cells/mm^3^ did not influence the MBL plasma concentrations, because the medians remained smaller in the coinfected patients in relation to the monoinfected patients. However, considering the use of a continuous numerical scale, it was possible to establish an inverse correlation between the plasma MBL concentrations and the HIV viral load in the coinfected and monoinfected patients, suggesting that MBL plasma concentrations might also modulate coinfection.

Other factors may influence the MBL concentration, such as polymorphisms in the *MBL2* gene, resulting in defects in the polymerization of the molecule, leading to functional deficiency and protein expression and reducing the activation capacity of the complement system [42–44]. Furthermore, the drugs used in the treatment of infections, such as those of ART [45–47], are metabolized in the liver and may affect the concentration of MBL because this protein is mainly synthesized in the liver [26].

Our research has some limitations, such as the absence of investigation of the HHV-8 latent or latent cycle, quantify of the HHV-8 viral load, evaluate functionality of MBL by measuring the activity of MBL in the complement system through C4b binding or measure aspartate aminotransferase (AST) and alanine aminotransferase (ALT) enzyme levels in the blood as a measure of liver toxicity. We suggest that future prospective studies evaluate these variables by associating or correlating them with plasma MBL concentrations in HIV/HHV-8 coinfected patients, especially in those who developed KS.

## Conclusion

Therefore, although the plasma concentrations considered deficient of MBL were not associated with HHV-8 infection in PLWHA, the coinfected patients had lower concentrations of MBL and an inverse correlation with the HIV viral load, suggesting that MBL might also be modulating HIV/HHV-8 coinfection.

## Methods

The aim of this study was to verify the association of MBL deficient plasma concentrations in HIV/HHV-8 coinfected and HIV monoinfected patients and correlate the concentration with the HIV viral load and CD4 counts in both groups.

### Design and study population

**Fig. 4:**
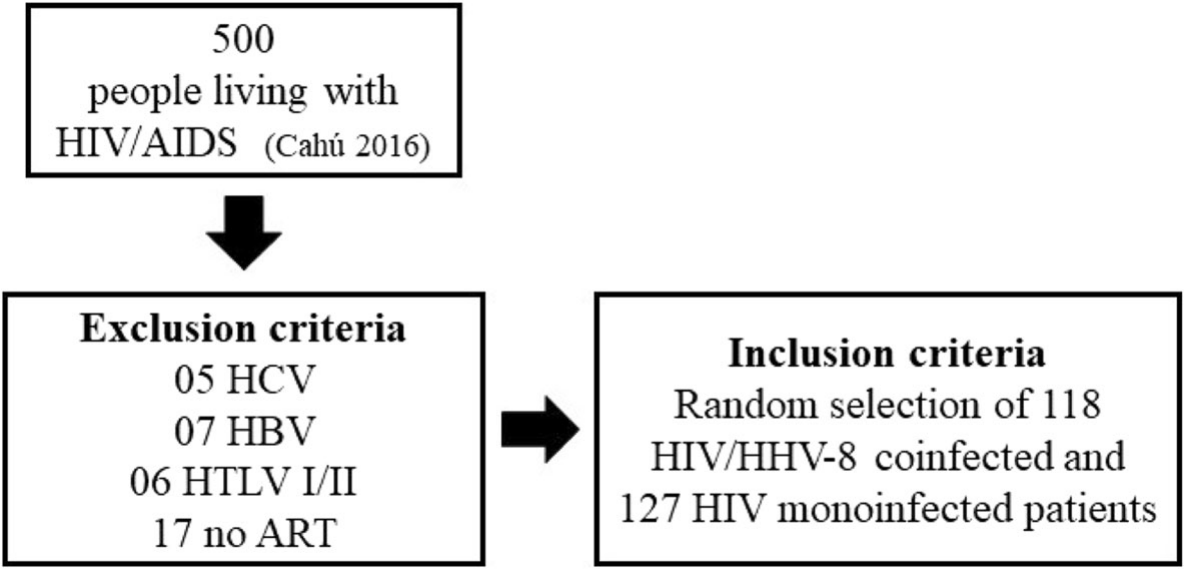
Participation flow diagram along with inclusion and exclusion criteria used in the research

This was an analytical study of case-controls, consisting of PLWHA that were monitored at the medical outpatient of Infectious and Parasitic Diseases of the clinical hospital in the Federal University of Pernambuco. Controls were defined as individuals with serological diagnosis of HIV infection, established according to Administrative Rule N°. 151, of October 14, 2009 (BRASIL, 2009), being characterized as HIV monoinfected. The cases were defined as PLHA with the serological diagnosis of HHV-8 infection detected by in-house enzyme-linked immunosorbent assay (ELISA) produced by Brazilian laboratory (Virology Unit of the Institute of Tropical Medicine of the University of São Paulo). Antibodies were identified against structural and non-structural proteins of HHV-8, being characterized as HIV/HHV-8 coinfected, according to Cahú et al. (2016). We excluded PLWHA infected by HBV, HCV, HTLV I/II and those not under ART¸ according to Fig. 4. The study was approved by the Research Ethics Committee of the Federal University of Pernambuco (number: 22428813.5.0000.5208) and all patients gave written consent to participate in the research, in accordance with the ethical, consent and permission rules.

### MBL plasma concentrations

The plasma concentrations of MBL were obtained by an enzyme-linked immunosorbent assay (ELISA) using a commercial *Human Mannose Binding Lectin* kit (MyBioSource, Inc.), with a detection threshold of 0.05 ng/ml. The samples were diluted at 1:100, and the protocol for the ELISA was performed following the manufacturer’s guidelines. The readings were performed on a spectrophotometer (Thermoplate®) with a wavelength of 450/630 nm. The plasma concentrations were considered deficient when they were < 100 ng/ml [18, 19, 25].

### Statistical analysis

To evaluate the deficiency of the concentrations, we used the odds ratios (ORs) and 95% confidence intervals (CIs), and the Mann-Whitney test was used to associate the MBL plasma concentrations in the HIV/HHV-8 coinfected and HIV monoinfected patients. The Spearman test was used to correlate the plasma MBL concentrations with HIV viral load and CD4 count, and these variables were included in the statistical models as units transformed into log_10_. For the statistical analyses and the construction of the graphs, we used GraphPad Prism software version 6.1 (GraphPad Software, USA) and Epi Info version 7.1.5 (CDC, Atlanta, GA, USA). Statistically significant values were indicated by *p* < 0.05.

## Abbreviations

ART: Antiretroviral therapy
CIs: Confidence intervals
CMV: Cytomegalovirus reactivation
ELISA: Enzyme-linked immunosorbent assay
HBV: Hepatitis B virus
HCV: Hepatitis C virus
HHV-2: Human herpesvirus 2
HHV-8: Human herpesvirus 8
HTLV: Human T-cell leukemia-lymphoma virus
KS: Kaposi’s sarcoma
MBL: Mannose-binding lectin
PLWHA: People living with HIV/AIDS
